# Report of the Metropolitan Commissioners in Lunacy to the Lord Chancellor. Presented to Both Houses of Parliament by Command of Her Majesty

**Published:** 1845-04

**Authors:** 


BRITISH AND FOREIGN

MEDICAL REVIEW,

FOR APRIL, 1845.

### PART FIRST.

### Analytical and Critical Reviews.

### Art. I.

*Report of the Metropolitan Commissioners in Lunacy to the Lord Chancellor. Presented to both Houses of Parliament by command of Her Majesty.—London*, 1844.

The historian, looking back at certain marked men, pronounces them to have been "before their age." They have seen clearly certain great truths, and have ardently, perhaps, promulgated them, but the world heeded them not; it took no great notice of the men, and less of their doctrines. But the time comes when their very truths become of deep interest to multitudes. The truth which seemed to be the property of one man becomes almost suddenly the inheritance of all. It would seem almost as if some spiritual power influenced at once multitudes of thinking and active souls, compelling them to believe unanimously, and giving them the willingness to act; like the invisible and universal influence of the spring season, which in a few weeks brings into action that life which has been hid in small unnoticeable seeds, or in dry naked twigs, branches, stems, and trunks; or in eggs or ovaries, or torpid in sleep; and in a few weeks clothes the fields and woods with a living garment, peoples them with living creatures, at once converting the lifeless and dead surface of the earth into a field for all forms of beautiful and vigorous life. It would be well if truth alone had this universal power over men's minds. Error may have the same mysterious influence. Whole nations may act simultaneously with such ruinous folly, that, as Bishop Butler said, when deeply pondering some sad facts of history, we can only account for them on the supposition that multitudes of human beings become at times simultaneously mad. These changes in public opinion are very curious phenomena. We are now in the midst of such a change; not one in which any insanity can be suspected, as it is a change based on the firm foundations of physical science, in accordance with the purest truths of moral philosophy, and of its highest form, Christianity, and leading to the most beneficial issues. May it be carried out with the energy and the earnestness with which nations have so often and with such self-sacrifice executed their errors. The "condition of England" question has now got hold of men's minds. The mere political condition, which within a short time so fiercely agitated them, has given way to the consideration of the social condition of the country. The ground of anxiety is not now whether the power of voting shall be given, or shall be withheld, from the poor man, in order that this or the other party in the state may be supported or weakened; but whether or not his actual bodily and mental condition shall be improved. Whether it is not the duty of the rich man to see that the tenement of his poorer brother is less crowded, better ventilated and drained, white-washed, wind and water tight. Whether it is not the duty of the whole community of the state to regulate the building of cottages, the draining of streets, the supplies of water, in order to prevent the wholesale causes of disease, which sweep away or weaken so large a number of our labouring classes; and whether improvement in the physical condition of the abject poor must not go hand in hand with attempts to raise them from their spiritual degradation. That the public are becoming interested in such subjects, the importance of which we have *known*, and not merely heard of, is most gratifying; for, as medical practitioners, we have watched men in their prime die of fever, poisoned by malaria generated in their close and filthy tenements filled with the destitute, or rising out of stagnant ditches or badly-made sewers; and we have made our way through the filth allowed to accumulate in the poor man's street, and have ourselves been half suffocated by the stench of his tenement; and we have seen the curved spine, and handled the scrofulous joint, and listened to the cavernous gurgle in a human being's lung, and practice has proved what physiology affirmed, that destitution and debilitating influences weaken the mind, rendering the individual an easier prey to vicious habits, and less capable of virtuous self-control. To our profession it is therefore of especial interest that matters have taken this turn; that newspapers, journals, and all ephemeral literature teem with discussions on the sanitory condition of the poor; that baths and washhouses are erecting for the labouring classes on a large scale; that Parliament is investigating the state of the large towns and cities, with the especial object of devising some means for the mitigation or prevention of those several causes of disease which are the result of a condition of things which is remediable by a paternal government.

One of the proofs of this change in the direction of the aims of government is the Report before us. It is an inquiry into the state of the lunatics confined in asylums in England and Wales, by a body of Commissioners, consisting of members of Parliament, men of business, barristers, and physicians, who were empowered by act of Parliament to visit all the lunatic asylums in the kingdom, and to investigate minutely the construction of the buildings, and to point out their advantages and defects; to examine their management, resources, superintendence, and domestic economy; to inspect the bedding, clothing, and food of the patients, and to inquire into the coercion which is used, the means of punishment, the occupations and amusements provided, and, in fact, every circumstance connected with their subsistence and general management; the mode in which the present laws and regulations concerning lunatics worked, and to suggest ameliorations. This Report is the fruit of their labours. Lord Ashley is their chairman, and the medical members are Drs. Turner, Bright, Southey, Hume, Waterfield, Bisset Hawkins, and Prichard, all of whom (except Dr. Bright) have signed the Report, which, from the weight of names of men in high estimation, becomes an important document. But though important, we fear it will be read by few. To readers in general there is no book more repulsive, within and without, than a Report. The narrow strip of letter press, the marginal references, the long quotations of evidence, strung together with common-place reflections, the object of which seems to be to make out a case, to show that the secretary has done something, or that certain commissions have not been altogether vain, or that large sums which John Bull has subscribed have really not been merely for the support of the officials (although much of it is thrown away in thus attempting to prove the contrary): the first look of the book, in its unmistakeable blue or olive paper covers, is at once with many sufficient to pronounce it "a sham," and to condemn it unread to all the base uses of waste paper. Now and then physiognomy deceives. *Nulla fronti fides.* A few grains of wheat are hidden in the bushel of chaff. Among the thousands of Annual Reports printed in this age of societies and commissions, it would be strange if there were not some which were profitable to others beside the printers, some exceptions to prove the rule; and as we think the Report before us is of this last sort, we purpose laying all that contains matter of importance before our readers in the present article.

It was probably owing to the Government not wishing to interfere with the Poor Law Commissioners, that this inquiry was not more complete. It will be seen hereafter that so many abuses exist in the lunatic wards of workhouses, that the investigation should also have extended to them, and that the Poor Law Commissioners themselves appear to be in ignorance as to the true state of these pauper lunatics (9336,) which are under their care.

### I. KIND OF ASYLUMS.

*County asylums for paupers.* These are erected and paid for by county rates. The great expense of their construction has prevented the erection of others. Some have cost as much as 200*l.* per head for those they will accommodate, whereas the average expense of a union workhouse is 40*l.* a head. Half of this large expense seems to have been caused by making the buildings fire-proof, and from the number of separate cells. The Commissioners object to any architectural decorations; they doubt the necessity of fire-proof buildings, as neither hospitals nor private asylums are fire-proof; and they question the desirableness of separate sleeping cells, as they find the rule is in private asylums for four or five to sleep in separate beds in the same room. We do not agree with the Commissioners, but with Dr. Conolly, in this latter particular. (See Dr. Conolly's Report on the Asylums of Paris, in our last number.) These asylums should be placed on high ground, on a dry soil, with cheerful prospects, where there is a plentiful supply of water, and proper drainage, surrounded by sufficient land to give outdoor employment and exercise, and not overlooked. The interior should be light and cheerful, and there should be light and warm galleries for exercise in bad weather. Some have these advantages, but in many they are partially or wholly neglected. The want of an ample supply of water in places where numbers are collected (many of whom are extremely dirty in their habits) is obviously a serious calamity, and the want of sufficient land is a great defect, as out-door employment (especially for the poor,) is a great means of restoring the health of the mind, and of promoting tranquillity. Warmth, dryness, and ventilation are most important. At Stafford, since the adoption of an improved mode of ventilation and warmth, dysentery which was before prevalent, has not occurred, and the same disease has been removed in the Dorset Asylum since the floors which were damp have been taken up and relaid.

In many of the older asylums the circulating steam or hot water apparatus has been introduced instead of open fires and stoves. In the Chester Asylum open fires in the day-rooms are still used "with a view to the enjoyment of the inmates, who much preferred them to a heating apparatus as being more cheerful." In the Kent Asylum, arrangements for ventilation (as should always be the case,) were executed with the building. The fresh air passes into a chamber beneath, where it is heated by passing over hot water pipes, thence it enters the galleries in a large volume near the ceiling, and it is drawn off through holes in the floor into air drains communicating with fires in the cellar, which fires are entirely supplied by the vitiated air from the galleries and dormitories. This is said to answer perfectly. It is obvious, however, that a decided error has been committed in allowing the air to enter at the top and drawing it off from the bottom of the rooms, as the air vitiated by respiration is thus necessarily breathed over again, which it ought never to be.

From the facts given, as well as from our own experience, we conclude that in day-rooms open fires properly guarded are preferable from their cheerfulness; for where low spirits and gloom and melancholy are the diseases of so many of the patients, every means should be taken to promote an opposite condition, and the mere question of expense should not decide the point. But refractory wards, galleries, and dormitories should be warmed by hot air, or by some system which combines warmth with proper ventilation, and in all new asylums this should be a part of the plan of the building, and not an after thought. Ventilation of the cells for the dirty patients, the Commissioners found often much neglected, and in some asylums the galleries which were not warmed artificially were extremely cold and comfortless.

Sleeping-rooms should be built on one side only of a gallery, as they make it gloomy when on both sides ; or if this latter arrangement is unavoidable, as in old buildings, recesses should be left between the rooms at intervals, lighted with windows in the outer wall. Light, if possible, should be admitted at the ends of galleries. The Commissioners are of opinion that good-sized sleeping-rooms, with a few beds, are preferable to single cells, as having a more free circulation of air, a more even temperature, and greater cheerfulness. We, however, consider a certain proportion of cells most desirable. Rooms on basement floors as a rule should not be used for lunatics, as they are cold, dark, and ill-ventilated. In some older asylums their cheerfulness has been improved by making them to open on green grass slopes instead of areas.

The Commissioners incline to the opinion that galleries should be used for exercise only, and not as day-rooms, as the preparation of meals, placing and removing benches and tables interfere with the chief object. There is, however, another point at which this may be viewed. These preparations and changes make a break in the day which cannot be otherwise than agreeable to these poor idlers in wet weather. And we suppose it must be for some such practical reason as this that the galleries in the Kent Asylum, one of the best, are used as day-rooms, and that the resident physician of Hanwell approves of their double purpose.

The yards should have as much light, sun, and prospect as possible. Many in the older asylums are gloomy, from high walls, and neighbouring buildings. Every yard should have a shed for shelter from the sun.

The Commissioners think that no asylum can contain more than 200 patients with benefit to themselves and to the public, from the difficulty of maintaining order and discipline in these establishments where so many servants are required. There must be of course a limit beyond which it is imprudent to pass, but the grounds for limiting the patients to 200 are not given. We agree with Dr. Conolly in thinking that 400 should certainly be the extreme limit. Eight out of the sixteen county asylums contain more than 200 patients, and in five of these the system of non-restraint has been successfully carried into effect.

By law the magistrates at the quarter sessions have the power of electing from their number a committee of visiting justices to superintend the internal management of county lunatic asylums, and to elect and dismiss officers and servants. This latter power seems often delegated to the medical superintendent and the matron; the Commissioners regard this as an abuse. But those who are conversant with committees of charities know that usually they are the outward and visible sign of the will of some single individual, who devotes himself to the business of the Institution, understands it, and really regulates it. And if there is no such person on the committee, no persons are better qualified than an able medical superintendent and a matron, for estimating the value and merits of the servants employed. A visiting magistrate, capable and willing, who devotes his time to an asylum, would get all the influence the law gives him; but such men are scarce, and cannot be created out of any body of magistrates.

A judicious suggestion is made that a general body of rules for the management of *all* county asylums should be drawn up, with uniform and concise tables for statistical returns. In doing this it would be advisable that all the printed rules of the different asylums should be collated by some physician practically conversant with the subject, and the rules chosen should be submitted to a barrister for revision, as no non-legal person is competent to judge of the precise bearing of any such form of words.

Rules, the Commissioners suggest, should be laid down for the management of those paupers who have no home or parish on leaving the asylum. There were in 1842, in the County Asylum of Lancaster, 118 of this class, and in 1843, there were 126 at Hanwell. Funds for this object have been left to several hospitals by charitable persons, an object well worthy the attention of the humane. The Adelaide Fund at Hanwell for this purpose is a most valuable institution. The Middlesex magistrates do not now adjudge a patient to Hanwell without a minute investigation of his claims by the county solicitor, and thus they benefit the pauper by discovering his settlement, and at the same time serve their own county. Every county asylum containing 100 beds or more is required to have a resident medical officer; and the Commissioners recommend in addition a visiting physician.

They further suggest that although a few of the county asylums are well adapted for this purpose, and a very large proportion are extremely well conducted; yet that as some are unfit for the insane, some placed in ineligible sites, some deficient in means of out-door employment, some defective in internal accommodations, some are cheerless and some too large, and in addition all expensive in their original construction,—that it is deserving consideration whether the power of erecting public asylums should not be regulated by some authority independent of the magistrates with whom it now rests. The great practical evil seems to be the expense of building new county lunatic asylums, which hinders their general adoption, and certainly few methods can be more likely to entail expense than the committing such a matter to a committee of magistrates, none of whom may be sufficiently acquainted with this kind of business to check in any measure the rapacity of architects, surveyors, or contractors, at whose mercy even a good man of business must be to a certain extent. The submission of plans and estimates to some competent tribunal, wholly independent of local interests, would be most desirable; but this is not a medical question, although as medical men we are much interested in it.

2. *Mixed county asylums.* A few asylums were built under an act, the intention of which was to support the paupers partly out of the payments of the rich by admitting private patients also. But the object has failed, the paupers in such establishment are less advantageously situated, and their maintenance is as expensive as in asylums established solely for their use.

3. *Military and naval hospitals.* The military hospital at Fort Clarence requires great improvements. That part of the naval hospital at Haslar set apart for lunatics is admirably adapted for its purpose. The rooms are spacious, lofty, and airy, with a fine view; there are excellent exercising grounds betwen the hospital and the shore, and the patients are frequently taken out in boats.*

4. *Public hospitals supported wholly or partly by voluntary contributions.* In the lunatic ward of Guy's Hospital, and to a certain extent in the Bethel Hospital at Norwich, the patients pay nothing; but in the others (seven are mentioned,) the patients pay the whole or the greater part of the expense of their own maintenance and medical attendance. "Private patients, who have been in better circumstance, derive much benefit from the comforts and advantages which these institutions supply at a moderate rate of payment." Among them the Commissioners mention with almost unqualified approbation, and they are by no means great praisers, the Retreat near York, and the Asylum at Lincoln, the Warneford Asylum near Oxford, and that at Northampton. The situa-

* See our account of this asylum, vol. XVII, p. 285.

tions of St. Luke's, the Bethel Hospital at Norwich, and the Asylum at Manchester are bad.

5. *Licensed Asylums.* These are of three kinds: *a.* For private patients only. *b.* For private patients and paupers. *c.* Licensed parts of workhouses for paupers.

*a. Asylums for private patients only.* The Commissioners found many of these susceptible of improvement; but on the whole this class of houses were well conducted and calculated for the comfort and well-being of the inmates, and owing to suggestions from the Commissioners were gradually improving, though from their experience they were convinced that some of the houses which they now commend would become scenes of great abuses were it not for constant supervision.

The chief abuse is in the illegal practice adopted by some proprietors of taking patients without certificates on the ground that they are merely "nervous," some of whom are actually insane. "Every individual confined under certificates is examined by official visitors, whose duty it is to satisfy themselves not only that he is properly treated, but he is also a fit person to be detained; and such investigation is some protection against persons of unsound mind being induced to make dispositions of their property." The neglect of this practice is obviously objectionable.

*b. Licensed asylums admitting private patients and paupers.* In some asylums which have been private mansions the family of the proprietor and a few private patients occupy the mansion, and the paupers are consigned to the offices and outhouses: such are the houses at Lainston and Nursling in Hants; Bailbrooke, near Bath; Plympton, Devon; Derby; and Duddeston, near Birmingham.

Owing to the visitation of the Metropolitan Commissioners the houses at Bethnal-green, which were among the worst, now rank with the best asylums. At Dunston Lodge and Chester Asylum, notwithstanding official remonstrances, one bed is allotted to two men.

*c. Licensed parts of workhouses.* Those at Morda, near Oswestry, Salop, and at Stoke Dameril, near Plymouth, are extremely ill-suited for the purpose. At Kingsland, near Shrewsbury, the licensed part of the House of Industry contained from 80 to 90 lunatics, nearly all of whom were chained by their wrists to their beds at night! and this barbarous practice is only partially discontinued in consequence of remonstrance.

### II. ABUSES AND DEFECTS.

Under this head are classed those asylums which deserve "*almost unqualified censure.*" (p. 46.) They are eleven in number, viz. Haverfordwest; St. Peter's Hospital, Bristol; West Auckland; Wreckenton near Gateshead; Derby; Lainston and Nursling, Hampshire; Kingsdown House, at Box, near Bath; Plympton, Devon; Nunkeeling, near Beverley; and West Malling, in Kent.

The asylum at Haverfordwest is a disgrace to the age and country. It is an old jail, unaltered for its present purpose, out of repair and destitute of every comfort and convenience. On their first visit in 1842 the Commissioners found eighteen patients in two rooms, the only article of furniture in both day-rooms being a single table, the rooms paved, dark, and cheerless. The dress of the lunatics ragged, dirty, and insufficient, and not a single change either of body or bed linen in the asylum. Their beds of dirty straw, the only covering a dirty rug, or a scrap of blanket. In a cell, lighted by a grating, where the stench was so offensive that it was scarcely possible to remain, was a woman, fastened to a chair and entirely naked! No place for excercise, no attempt at employment or amusement, prayers never read, never visited by a clergyman, and scanty and unwholesome food. After strong remonstrances to the magistrates there was some improvement, but the place is still wholly unfit for the cure or treatment of the insane.

St. Peter's Hospital, Bristol, is entirely unfit for its purpose, as well as that of West Auckland, where, in a small, cheerless, unglazed room, were five men confined with chains.

At the Wreckenton Asylum, near Gateshead, in 1842, it was the practice to chain patients by their leg on their first admission "to see what they could do." The patients, and their rooms, and bedding were filthy, and chains were much used. Remonstrance had some effect, but the house is unfit for its purpose.

At Derby the beds were filthy: the paupers occupied what had been the coach-house and stables, the rooms low, comfortless, and unventilated. "An epileptic was in a bed in a dying state, and the windows and door of his room being closed, and there being no opening or other means of producing a free circulation of air, the apartment was most offensive. Some saw-dust had been thrown upon the floor to absorb the urine, but nothing had been done to purify the air." (p. 57.)

At Lainston, in Hampshire, the paupers were dirty and ill-clothed, and whilst the proprietor's family and female patients occupied the mansion, the stabling and outhouses had been converted into the residences for paupers. There were seven women with iron hand-locks and chains by day, who were also chained to their beds at night. The remonstrances of the commissioners have been attended to. At Nursling, Hants, the paupers, as at Lainston, were placed in the outhouses, which were dilapidated, and so ill-constructed that they could not be made comfortable. The bed-rooms for the dirty were offensive, confined, and had unglazed windows with only shutters.

At Kingsdown House, at Box, near Bath, the violent and the dirty were crowded together with the quiet and convalescent; and the noise and confusion were extreme. Many women were coarsely ironed, and one was chained by the leg to a seat in a paved passage to prevent her hurting herself in her fits! Eight or ten women were chained or strapped in their beds at night. The commissioners have made three annual visits, and notwithstanding their suggestions, it remains in the same unsatisfactory state.

But the asylum at Plympton, Devonshire, bears away the prize for filth and inhumanity. There were seventeen patients in a room seventeen feet by twelve, with no table, and seats for ten only. In a day-room was a young woman in a state of furious mania, six weeks after delivery, in a strait-waistcoat, chained by the arm and leg to a bench! Another woman in a strait-waistcoat was lying in a hole in the middle of the yard with her head exposed to the broiling sun. The dirty male patients slept in a damp, close room, which had been a dairy, with one small unglazed window closed by a wooden shutter. Twenty-one were chained to their beds at night. The night-cells damp, and dark as cellars; windows unglazed, floors soaked and reeking with urine, and partly covered with straw and excrement; filthy straw for beds, and in some (containing women,) there were no windows, but the light was conducted by a grating over the door opening into a passage, and so little of it that the commissioners in the day-time were obliged to use a lantern to get this sickening and shocking sight. And for each of these paupers 10*s*. 6*d*. was paid weekly, and one guinea on admission to this foul and loathsome den. The remonstrances of the commissioners have been useless, but through Lord Ashley, the matter has been taken up by the Earl of Devon. The asylums at Nunkeeling, near Beverley; and West Malling, Kent, were found in a disgraceful state. The latter has been since closed.

But we must condemn the system generally, quite as much as the conduct of individuals acting under it: that system by which pauper lunatics are made a source of profit to private individuals who embark in the lucrative employment of proprietors of lunatic asylums The house of a country gentleman is rented or purchased. The mansion is used for the proprietor's family, and his rich patients; whilst the offices, stables, and coach-houses are fitted up as rooms for pauper lunatics, on whom much of the profit of the whole establishment depends, as they consume the remains of the rich men's tables. They are the receptacles of superfluous provision which would otherwise be wasted. And as it is necessary to keep up a large establishment for private patients, whose numbers necessarily fluctuate, the certain income from a body of paupers is an additional advantage to the pocket of the proprietor. On this system they may be well fed, but they are necessarily ill-lodged; for it is impossible to convert offices and outbuildings into apartments fit for the insane, unless at an expense which would destroy the proprietor's profits. Thus classification, except in a wholesale imperfect way, is out of the question: there are no fit accommodations for the dirty class, as it is an expensive process to keep them clean: and as no adequate provision can be made for amusement and occupation the patients are dull, listless and idle; and, from want of occupation, restraint and coercion become almost inevitable. It is a common argument, that the accommodation provided in these outhouses is better than the pauper has been used to at home. This may be the case, but a cottage with its scanty conveniences may be fit for a sane man, but entirely unfit for an insane one: for whose care, perhaps, and most certainly for whose comfort, certain accommodations are essential, and for which the parish pays a much larger sum than they would allot as out-door relief to a pauper labouring under ordinary sickness; or for a lunatic who is so harmless as to remain with his friends: the average expense to unions of pauper lunatics and idiots who are provided for with their friends being 2*s*. 7¼*d*. a week, whilst the average weekly payment to licensed houses is 8*s*. 11½*d*. The remedy fortunately is known:—*County asylums for all pauper lunatics;* a remedy which we are happy in believing will, ere long, be universally applied.

The humane system of non-restraint, or even the system which allows occasional restraint, can only be carried out where arrangements can be made for classification, and for employment, and cheerful amusement; and these ends can alone be attained (unless for rich men) in large asylums expressly constructed for the purpose. The expense of building asylums is large, but, after all, no great immediate burden for a whole county, and the annual saving is so considerable, that no greater expense is entailed than at present, whilst the comforts of the paupers are greatly increased. Thus the average expense for paupers in the county asylums is 7*s*. 6$\frac{3}{4}$*d*. weekly, and in licensed houses 8*s*. 11$\frac{1}{2}$*d*.; a saving of nearly eighteen pence a week, which, in an asylum containing 300 patients, would amount to 1170*l*. a year. The rigid economy which is now enforced under the New Poor Law Act is an additional argument.

That eleven asylums in which such flagrant abuses exist are still to be found, and that in almost all these instances the commissioners' remonstrances, and the ordinary legal powers they could exercise, were unavailing to repress the abuses, are strong and unanswerable arguments in favour both of a more constant and strict supervision by competent persons, with increased powers given by law. The commissioners admit that the visiting justices, as a body, do their duty, but yet that they have met with three instances, in two of which the asylum had not been visited for one year, and in the other for two years and a half. There are some points in which the justices have never directed their attention, and are not competent referees; such, for instance, as the fitness of the lunatic for liberation, the nature and effects of the employment provided, with a view to diverting the disease; the character of the medical reports indicating the care and intelligence of the medical attendant, and the quantity and quality of food. But, besides this, there is no security that the justices themselves are competent judges of the cleanliness, bodily condition, and common treatment of lunatics, or of the fitness of the accommodation which is provided for them. From the time of Justice Shallow to the present "enlightened" period, justices of the peace occasionally supply specimens of pompous and ridiculous inanity, and monstrous ignorance, which verge on the incredible; and nothing which Shakspeare or Fielding wrote is a keener satire on their body than the certificate of the justices who visited the West Auckland Asylum, as dryly detailed in this Report.

We have mentioned the West Auckland Asylum as one of the eleven which "deserve almost unqualified censure." The commissioners visited it on the 5th of December, 1842. They found 13 men and 16 women shut up in two cheerless rooms, the violent and quiet, the clean and dirty together, with one small walled court only for exercise; so that when one sex were there, the other were locked up. In the men's room, which had one unglazed window, five men, although tranquil, were confined with leg-locks, and two of these, in addition, had iron handcuffs, and fetters from the wrists to the ankles. They slept two in a bed. One woman was leg-locked by day, and chained at night to her bed. The commissioners stated that it was *entirely unfit* for the insane. It was visited afterwards, but on the same day, by two magistrates. We can fancy the sleek, well-dressed, spotless, middle-aged gentlemen filling up the time between lunch and dinner by a ride to the Asylum on a bright, frosty December afternoon. They have left their houses in that perfect state of order and comfort which belong to those of good taste, ample wealth, and the hereditary habits of English country gentlemen. The interior arrangements combining luxury, comfort, order, with the utmost cleanliness, and even in this wintry day warmth with freshness; and the eye catches through the window, green lawns and shrubberies, and beyond, ancestral trees, noble even in their winter bareness, with deer on the greenest sward. It need not be said that the cuisine is excellent, and the arrangements for the table complete; and that the stables are as comfortable for the horses, and the kennels for the dogs, as the mansion for its human residents. The magistrates arrive at the Asylum, they go through rooms where disorder reigns paramount, where sickly men are crowded in a room with one unglazed window, and that without prospect; where those who have been too excited have been tranquillized and soothed by the restraint of heavy chains, in which they are still manacled, though quite subdued, lest they should be noisy again; where those who have been used to a life of the open air, and to whom an in-door life, on compulsion, is as irksome as it would be to themselves, and more so, as their disease is most tranquillized by bodily exercise, have one small walled court, to which they have only occasional access, until they say "they have been made so tender by confinement that their health was destroyed;" and having seen all this, the justices sit down and write thus:

"5 *December*, 1842.

"We this day visited the Asylum, and found that the commissioners had just left it. We found everything in good order."

*And after finding everything in good order*; and certifying to it, the carriage drives up, the justices, as it is colder, wrap themselves in fur cloaks, draw up the windows, get home in excellent time to dress leisurely for dinner; and four courses, and some very old port receive an additional zest from their consciousness that part of the day has been spent in generously fulfilling "unpaid" duties, and the justices really sleep as sound, and rise as contented as if they had harmlessly killed partridges the day before, instead of perpetuating for another quarter the miseries of thirty fellow-creatures. Good-natured, and honest men doubtless in their way, but as incapable as visiting justices, as those worthy constables Dogberry and Verges were in their sphere. And whilst neither ability, nor knowledge of the law is required as a qualification for the magistracy, but merely a certain standing in society, such exceptions must occur, and they are strong arguments in favour of some change in the present system of visitors. Mr. Tuke's sentiments on this point are well known, and give great additional force to the arguments against the present vicious system of visitation.

### III. CONDITION OF PAUPERS ON ADMISSION.

One of the special objects of the commission was to inquire into the evils which result from delays in sending paupers into asylums, evils applicable to a certain extent to private patients also.

The medical superintendents of all the County Asylums, except two, state that paupers are sent at so late a period of the disease as to prevent or impede their recovery. And we need not say that statistical returns prove that the durability of insanity is in proportion to the early date of admission. Tables of the cases at the Retreat near York, and at the Asylums of Lincoln, Northampton, Lancaster, Middlesex, and the West Riding of Yorkshire, exhibit the large proportion of cures when the patients are admitted within three months, the less proportion after three months, and the almost hopelessness of cure where lunatics are kept in workhouses or elsewhere for a twelvemonth before admission.

At St. Luke's, where recent cases only are admitted, the cures average 65 to 70 per cent. The delay is partly owing to the ignorance of guardians and overseers as to the importance of early treatment, and to their unwillingness to incur immediate expense; but it is chiefly owing to a want of proper accommodation. Three sets of tables in the appendix prove this sad defect unanswerably.

1st. In those counties where there are either county or subscription asylums, there are 8078 pauper lunatics, for 4215 of whom, or but little more than half, there is accommodation in the asylums, whilst there is none for 3843.

2d. In those counties of England and Wales having asylums there are 14,088 lunatic paupers, but there is accommodation in those asylums for only 7457 pauper lunatics, and no such accommodation for 6156.

3dly. In 21 counties of England and Wales there are no asylums, either public or private, consequently their insane poor (amounting to 2762) must be sent to a distance at a considerable expense.

But besides this deficient accommodation, the public asylums are crowded with a large and increasing number of incurable cases. In 15 public asylums there are 4367 pauper lunatics, 658 of whom only are curable, and 3709 incurable. Thus these asylums are becoming more and more receptacles for the incurable, rather than hospitals for the cure of insanity.

The county asylum at Hanwell is an instance in which these alleged evils, which are common to all, have from the size of the county increased with a rapidity unequalled elsewhere.

It was originally built in 1831, to hold 300 patients: it was subsequently enlarged to contain 350 more, but 1000 patients are now confined in it by using offices and basement stories for these purposes, for which they were not originally designed. The cost of land and building has been £160,000, and the expenditure £28,000 a year, and yet there are 429 lunatics in Middlesex who cannot be accommodated. No measures have been enforced by the magistrates (in fact they have no power to enforce such measures,) for selecting the more recent cases when vacancies occur, and the exchange of incurable for curable cases has been refused, so that the house is filled with old and incurable cases, which the resident physician attributed "almost entirely to the neglect of proper remedies in the early stages of the disease." In March 1844, there were 984 patients, of whom 30 only were reported curable, and 40 admissions appeared during the three months preceding.

The Middlesex magistrates refuse to exchange incurable cases for curable, on the grounds that the incurables were better fed, and more comfortable at Hanwell than in workhouses. And such a reason would inevitably actuate humane men who looked at men as individual human beings, not like political economists, on mankind in the heap, although such opinions are somewhat out of date, and obnoxious to many statists, commissioners, and reporters. And after all, this exchange is but a most inefficient mode of remedying the evil in question if such it be. The remedy suggested by the Commissioners is *houses of refuge for the incurable.* These, as they would not require the same medical staff, number of attendants, minute classification, and internal arrangements of lunatic hospitals, would be at the same time less expensive, and, what is of greater importance, would be the means of giving recent lunatics a chance of cure, instead of consigning them to hopeless lunacy, miserable to themselves, and a useless burden to the state. If this, or some other plan, for the speedy reception of recent cases, is not adopted, county asylums will be eventually such houses of refuge only, as the number of incurables increases as a matter of course every year, and as the greater the number of incurables in the asylum, the fewer chances there are of recovery for the more recent cases.

At first sight, the proposal of the entire separation *in different asylums* of the incurable from the curable, will probably meet with general assent. The plan, however, is far from being unexceptionable; and we entirely concur in the modified views entertained by Dr. Conolly, as so admirably stated in the following paragraph of his Report on the Asylums of Paris, which we make no apology for reprinting from our last Number:

"When I contemplate this, the worst class of incurables, I always think with concern of the disposition apparent in some quarters to make a formal separation, in distinct asylums, of the *curable* and *incurable*. Such propositions are abstractedly pleasing; but the effect ought to be well considered. In the case of many incurables, the manners and habits during a great part of every year are superior to those of the curable, and, therefore, not detrimental to curable companions. The separation and transmission to an asylum for incurables would be felt by them as a sentence of eternal imprisonment. As it is, hope, the only blessing left, never dies with them; and some of them recover, after years of affliction, and beyond any ordinary expectation. At once to pronounce many cases incurable which seem so would be to commit many errors; and the asylum for those pronounced curable would always be half filled with cases really not to be cured. The only class, therefore, of the incurable who could safely be severed from the rest of the patients, as mentally dead, would be these poor imbecile, helpless creatures, fallen into fatuity and dementia: and there is much reason to fear that by separating them from a curative asylum they would lose all the benefits which alleviate their unhappy lot. It is quite unnecessary to mix them in wards with more intelligent patients; but it is surely humane to keep them under sufficiently active medical direction in an asylum where it is admitted as a principle, that if few cases admit of cure, every case admits of improvement."

The removal of incurable lunatics who have been some years in a well-conducted asylum to a workhouse is a different case altogether to the selection of the most recent cases for admission, in preference to the more confirmed, and it seems that no such care is exercised, but that the admission is indiscriminate, and without reference to the urgency of the case. The great expense of incurable lunatics is shown from the tables of Hanwell. It has been found that lunatics who recover generally do so within the first year, and after two years the chance of recovery is very small. Out of 936 patients in Hanwell, 696 have been there more than two years, and were reported incurable, and the average duration of the confinement of each had been 6 years and 9 months. As each patient at Hanwell costs the parish 22*l.* 4*s.* annually, each of of these 696 patients will have cost his parish in this time 140*l.*; not including the support of the families of many of them.

The Commissioners direct attention to the printed notes of the Poor Law Commissioners in which it is stated that "the Poor Law Commissioners believe most of the persons of unsound mind detained in workhouses are incurable harmless idiots. The Commissioners of Lunacy were not empowered to visit workhouses, but in 1842 they visited such as lay in their road, and obtained the Lord Chancellor's permission to visit others. The particulars of four are given. At Redruth workhouse in Cornwall there were 41 insane paupers, of whom 6 only were idiots; several of the lunatics were violent. At the union workhouse, Bath, there were 21 lunatics, and 2 under restraint. In the Leicester union there were 30 lunatics, 12 of whom were dangerous. In the workhouse at Birmingham there were 71 insane persons suffering under all forms of the disease.

These instances show the ignorance of the Poor Law Commissioners on the state of pauper lunatics in their own unions. But besides this ignorance of facts, the reporters object to the mode in which the Poor Law Commissioners construe a doubtful clause in the Poor Law Amendment Act, so as to give their sanction to the detention of lunatics in workhouses provided they are not dangerous, without adverting to the probability of their being curable, or not; a recommendation highly objectionable, as leading to the belief that there is no harm in keeping lunatics away from asylums as long as they are not dangerous, and thus producing an incurable condition. Other objections to lunatics being confined in workhouses are that they have neither separate wards, airing-grounds, medical men skilled in the treatment of insanity, nor experienced attendants, all of which are required. Even congenital idiots are not harmless, as they often exhibit disgusting and depraved propensities.

The ignorance of the Poor Law Commissioners on the state of pauper lunatics in union workhouses, the mistakes they are of course liable to in judging of points requiring medical knowledge, and the large number of pauper lunatics (between six and seven thousand) confined in workhouses, show that it was a serious omission in the act that the Commissioners of Lunacy were not empowered to visit workhouses as well as licensed asylums; and furnish additional arguments for a system of efficient visitation of all establishments containing lunatics by competent persons under the authority of government.

IV. FORMS OF DISEASE, MEDICAL TREATMENT, DIET, AND CLASSIFICATION.

We need only allude to a sketch of the various forms of insanity with which this part of the Report commences, and which is drawn up for non-medical readers. It is clear, simple, and practical. The *medical treatment* of the insane was an object of careful inquiry, and although the commissioners often found difficulty at getting at the truth, yet on the whole they trust they have gained a tolerably correct knowledge of the means employed. They found great diversity of treatment. In some exclusion and safe custody seem to be the sole objects, no plan of cure being attempted. For this a resident medical man, or one very near, is necessary. Many asylums having less than 100 patients are under the management of persons ignorant of medicine, and are visited by a medical man at a distance. Several of the private asylums are the property of physicians who reside in them, and many of these appear to treat their patients with judgment and skill. Amongst the most frequent causes of insanity in paupers are habitual intemperance, poverty, grief, disappointment, want of sufficient sustenance—all tending to weaken and exhaust the body, a condition often aggravated by sending them to workhouses, instead of to asylums, where they might have at once medical treatment. The general opinion of the best informed medical attendants is that the most successful method of cure of pauper lunatics is to remove this state of bodily weakness and exhaustion, by a restorative plan calculated to reproduce a vigorous state of bodily health. For this purpose are employed a nutritive and tolerably full diet, consisting of a considerable proportion of animal food, wholesome bread, milk porridge, a milk thickened with various farinaceous substances, and good broth, with a moderate quantity of malt liquor, ale or porter, and in some extreme cases wine or other stimulants. Warm clothing and bedding, and a moderately warm and dry atmosphere, are indispensable in promoting the cure and comfort, as for the most part the circulation is languid, and suffering arises from exposure to cold and damp. "Exercise in the open air in cheerful airing-grounds: baths, either warm or cold, according to the state of the circulation, and the habitual temperature of the skin; frictions promoting cleanliness and dryness of the surface of the body, and tending to keep up the action of the blood-vessels to a certain healthy standard, are generally found to promote the restoration of patients whose cases are of a curable description." These are often assisted by tonic and stimulant medicines. The whole of this plan is said to prove beneficial only when there are no signs of congestion of the brain, or tendency to epilepsy or paralysis: topical bleedings and counter-irritants are then necessary. The tonics most used are carbonate of iron, cinchona, sulphate of quina, gentian, combined with aloetics if there is costiveness, or with astringents when in cases of great debility there is diarrhea or dysentery. A moist or relaxed state of the skin, with cold extremities; a shrunk or shrivelled surface, with a livid and blotchy, or pale and yellow complexion, and feeble circulation, frequently coexist with insanity arising from depressing causes; and great advantages are said to arise from carbonate of ammonia, in frequent doses, for a considerable time. Emetics and powerful purgatives, unless indicated by any temporary disorder, are said in this form of insanity to be injurious. It is the testimony of the best informed medical superintendents that the restoration of bodily health is accompanied with marked improvement in the state of the mind. There are, as may be supposed, some medical superintendents who do not either practise or agree to this very judicious system.

The Commissioners believe that an ample supply of sufficient food is one of the most important aids towards the relief or cure of insanity among the poor. They have been assured by various intelligent superintendents of asylums that insanity has been frequently alleviated, and repeatedly cured, solely by increasing the quantity of wholesome food; and it is in consequence of their opinion that the amount of food allowed to pauper lnnatics, and the rate of payment made for them in private asylums, which is sometimes too low to remunerate the proprietor for a more liberal supply, should be under the control of official visitors. External warmth is very necessary in this debilitated state. Exercise is very important, but for the most part pauper lunatics are shut up in small cheerless yards.

There is a difference of opinion as to the use of opiates. Some abjure them, others look upon them as most valuable in cases of agitation and excitement, and have recourse to them on all occasions when want of sleep and restlessness produce debility and exhaustion. "This last practice seems to be gaining ground. Preparations of opium and other sedatives given in repeated and sufficient doses are thought by the best informed practitioners, who conduct the medical treatment in the large asylums, to be of great efficacy in subduing excitement and agitation, and conjoined with the use of baths, cold applications to the head, antispasmodics, and aperients, are said to promote the cure of mania in the early and acute stages." Several instances are recorded, at the licensed houses of Bethnal Green, of patients having been restored to reason, in a very short time, by the skilful administration of opiates.

These observations on treatment apply principally to recent cases, in chronic ones medicine is of less efficacy, but much may be done by skilful medical and moral management.

*The classification* of lunatics is one of the most important ameliorations in their management which has been introduced of late years. The principle consists in distributing them according to their mental disorders, and in associating those whose intercourse is likely to be mutually beneficial, and in separating others who mutually annoy and irritate. The rules have been entirely founded on experience.

1st. The first object is the separation of dangerous lunatics from others, keeping them in one or more separate divisions, surrounded by a sufficient number of experienced attendants.

2d. Restless and noisy lunatics require separate wards and airing-grounds, removed as far as possible from those of a quieter sort. In some asylums, where there is no such arrangement, there is an appearance of restless agitation among all the patients.

3d. Dirty patients, such as fatuous persons, insensible to the calls of nature, require a separate department.

4th. The classification of melancholy patients requires great discrimination. They are often aware of the state of others, and their own condition, and contemplate with horror the prospect of being reduced to the plight of demented or violent maniacs, if associated with them; so that they should not be placed with those whose intellects are more deeply injured than their own. They occasionally derive benefit from the comparison and contrariety of their several illusions, and are cheered by associating with patients of a lively and excitable habit of mind. From their suicidal tendency they require careful watching. In the Lancaster Asylum the suicidal patients are associated with the cheerful, and with advantage.

5th. Epileptics should be kept apart. Their fits alarm the timid, and they themselves are injured by the excitement of the noisy. At Lincoln they are placed in dormitories, where an attendant, who is placed so as to have a complete view of the apartment, watches them throughout the night; a plan deserving imitation.

6th. The tranquil and convalescent are placed together.

The Gloucester Asylum is a fair specimen of the classification in county asylums. The different classes are as follows: *a*, quiet patients and those approaching convalescence; *b*, the epileptics; *c*, the fatuous; *d*, the dirty and noisy; and *e*, the working class, forming a distinct body, consisting of convalescents, and some incurable patients who are yet capable of cultivating the garden and grounds.

The classification of the Lancaster Asylum is the most complete. On each side are ten wards. 1. Cases of dementia, associated with active, orderly, and quiet cases, who have been some time in the house, and can render the demented assistance. 2. Recent cases, associated with orderly and active ones as above. 3. Patients who have not manifested a tendency to violence, to suicide, or to escape. 4, Convalescent cases, a few old cases, and one or two suicidal cases. 5. Refractory and excited cases. 6. Suicidal, with cheerful and watchful cases. 7. Refractory patients, and violent epileptics. 8. Epileptics who are not violent. 9. Aged quiet ones of long standing, and a few suicidal cases. 10. Infirmary cases.

IV. OCCUPATION, AMUSEMENTS, AND EXERCISE.

There is no difference of opinion as to the importance of occupation in insanity; improving the bodily condition, and diverting the mind from painful or delusive trains of thought. In most instances it is desirable that the patient should be enabled to follow the occupation to which he has been accustomed ; or, if he has not been brought up to any regular pursuit, that he should be instructed in one to engage his attention. Gardening and agricultural labour should be provided, as they combine employment with out-door exercise: but, as regards pauper lunatics, their health should be the sole consideration, and not the profits of their labour; and thus moderate labour only, to strengthen and not to fatigue the body, is desirable, and such as will afford amusement without harassing the mind. A farm and extensive gardens should be attached to every large asylum. Music, dancing, and games (especially in the open air) are useful, except where the patient is too excitable : and books are important helps in promoting a serene and happy state of mind; and especially those of a cheerful kind, where the patient is melancholy. The Commissioners have found in most asylums numerous religious works, and sometimes few besides; and they have urged the necessity of procuring entertaining books adapted to the patients' capacities. This we think very judicious advice. By amusing books (as Bishop Butler says,) "time, even in solitude, is happily got rid of, without the pain of attention ; neither is any part of it more put to the account of idleness, we can scarce forbear saying, is spent with less thought, than great part of that which is spent in reading." This, though it may be an objection to the inordinate reading of amusing books by the healthy, is an argument in their favour for the insane, who are incapable of much thought, or of much attention, but who require some amusing occupation which requires no great mental effort.

In the better conducted asylums, books are placed at the disposal of the patients; trades and other in-door occupations are encouraged, in some cases rewarded; and the patients regularly labour, in the proper seasons, in large gardens and farms. In the Wakefield Asylum, which has a garden of three acres, and a farm of forty acres, there were in Sept. 1842, 398 patients, 255 of whom were employed. They belonged to a manufacturing district, and occupied themselves in woollen and cotton weaving, and all the clothes and shoes of the inmates were made by them. They made fancy articles for sale, and performed all the gardening and agricultural labour. Amusements were provided, which with their occupation were highly beneficial. In the Dorset County Asylum they are encouraged to occupy themselves by an extra diet. The Kent, Dorset, and Gloucester County Asylums, and Dr. Warburton's at Bethnal Green, are mentioned as excellent in these respects. But in many there is a great deficiency, not giving the lunatic a fair chance. We have before alluded to the close and small yards, and the want of airing-grounds in many asylums; besides these the Commissioners occasionally found the patients without occupation, books or amusement.

*Restraint.* One of the especial objects of this commission, was to inquire into the whole subject of coercion; and the Commissioners have made minute inquires as to each individual found under restraint,—and as to the system adopted in every establishment, in this respect.

They found two courses adopted. In some asylums the disuse of restraint was the rule, but it was used in cases of necessity. The usual forms of mechanical restraint are strong dresses, strait-waistcoats, muffs, gloves, straps, leather or linen belts; and as has been mentioned, for paupers in some cases, iron leg-locks, handcuffs, and chains. In other asylums "the superintendents and proprietors state that they manage their patients without having recourse to any kind of restraint whatever;" but they employ manual force and seclusion.

"It is said by these persons, that when any of the limbs are confined by the strait-jacket, the belt, or by straps or gloves, he is under restraint; but in cases where he is held by the hands of attendants, or when he is for any excitement or violence forced by manual strength into a small chamber or cell, and left there, it is said that restraint is not employed. In those cases where the patient is overpowered by a number of keepers holding his hands or arms during a paroxysm of violence, it is said that there is no mechanical restraint. Here restraint of some sort or other is manifest; and even in those cases where the patient is forced into a cell by manual strength, and prevented from leaving until his fit of excitement shall have passed, it is difficult to understand how this also can be reconciled with the profession of abstaining from all restraint whatever, so as to be correctly termed 'non-restraint.' It seems to us, that these measures are only particular modes of restraint, the relative advantages of which must depend altogether on the results." (p. 138.)

If this was a mere verbal criticism as to the use of the terms restraint and non-restraint, we should have no objection to it, although we should object to the sarcastic way in which it is put; but the impression conveyed by the whole is, that there is no great difference in these two modes of management, although one is called by a more popular name than the other; and on this point we wholly differ. A man instantly overpowered by the superior bodily strength of several others may at the time be irritated, but he does not feel degraded, as one who is placed in mechanical restraint: and this makes a vast difference. The Commissioners leave out of consideration the fact that a human being is dealt with, one with a sense of shame and honour, with strong likes and dislikes, passions, and feelings, often rendered more acute, susceptible, and exaggerated by mental disease. On such a question we may unanswerably appeal to a man's own feelings, and ask each reader whether it would not be to him the least evil to be overpowered by the manual force of many, and secluded, than confined by belts or handcuffs in the presence of others. The manacled lunatic may be quiet, but is he not brooding on the indignity, and in sullenness encouraging revenge? But let us take a picture of the effects on the patient's mind under mechanical restraint, drawn from the life, by one who had long lived among such scenes. "The indignation of those on whom restraints were forcibly imposed; their eloquent exposition of its degrading and maddening effects; their fierceness in running about the wards in a strait-waistcoat; the alarm and discontent their presence occasioned to other patients; their speechless joy, their sobs and tears, when unexpectedly liberated, when both in seclusion and restraint, in the midst of a paroxysm of reproaches prolonged until they were covered with perspiration, and their lips were literally covered with foam, are things of which the memory can never be effaced from the mind; and they make the ear deaf to specious representations of the comforts of straps and strait-waistcoats."*

This is not therefore a mere question of words: the difference is not merely as to the degree or quality of the force, but as to its effect on the mind of the human being; and as one of the modes produces a mental impression different from the other, the two should be considered (as they usually are) distinct and separate; and therefore, although the terms restraint and non-restraint may not be actually correct as mere terms, yet there is a distinct meaning attached to them, owing to their being used to express two very different proceedings.

But we continue the Report. The Commissioners are gratified in reporting, "that in every public and private asylum which is well managed, bodily restraint is not permitted, except in extreme cases, and under the express sanction of a competent superintendent. The unanimous opinion of all the medical officers and superintendents of these public and private asylums is, that the diminution of restraint in the treatment of lunatics has not only lessened the sufferings, but has improved the general health and condition, as well as promoted the comfort of the insane;" in which opinion they entirely concur. (p. 139.) The 11 asylums, whose names we have before given as grossly mismanaged, are not included, of course, in this commendation, as in many of them there was "an excessive and highly censurable degree of restraint," such as furnish good grounds for a refusal of their licenses.

The non-restraint system is steadily carried out at Lincoln, Hanwell, Lancaster, Gloucester, Suffolk, Northampton, and Haslar, and the superintendents think with advantage. The Commissioners found the asylums at Gloucester, Lancaster, Northampton, and Haslar very well managed, and the patients tranquil and comfortable. *"No inconveniences whatever have been experienced at Gloucester and Northampton."* In 1842 there were four deaths by suicide at Lancaster; but the superintendents stated that had mechanical restraint been in practice, it would not have been resorted to in either case. "The present medical officers at the Lancaster Asylum have carried out the system of non-restraint to its fullest extent. By every expression of kindness, by appearing to sympathise in the patient's imaginary sufferings, and by taking a deep interest in all his concerns, they endeavour to soothe morbid irritation, and thus allow an opportunity of restoring the healthy action of the mind, and in several recent cases with success." That the system of non-restraint is carried out at Hanwell, and with what glorious results, is too well known to be more than alluded to. Here, in 1843, the Commissioners found no patient under mechanical restraint, but they saw a violent female who endeavoured to bite others and herself, thrust by main force, and with great difficulty into a cell, where she threw herself against the door and struck it violently; during this scene there was much confusion. In another cell a woman nearly suffocated herself, by tearing a woollen rag, and making it into balls, which she swallowed. One woman ran violently against another and overthrew her. Another's arm was cut, from having thrust it through the window of her cell; and several patients had cuts and bruises. In their visit to Hanwell in 1844, they "found

* Third Report of the Resident Physician of Hanwell, 1841.

the asylum in good order, and the patients, with one or two exceptions, tranquil and comfortable, and not one under mechanical restraint."

We observe that the criticism of the Commissioners is chiefly confined to the female side of the house; and we suspect that an explanation of the fact of disorder being found there, may be found in the absurd system there sanctioned by the magistrates, of giving to the matron an immediate authority over the patients on this side of the house, without waiting for the sanction of the physician.

In the Suffolk Asylum all were tranquil except the ward of refractory females, which was a scene of distressing turbulence and confusion. On a second visit in the same ward they found some of the patients half-naked, from having destroyed their clothes; one was, during the whole time, struggling with the nurse; two of the most violent had just been removed; and the fury of the rest was excessive. Here also, we should expect, though we know it not, that there must be a divided authority in the establishment: some mismanagement there must be. At Lincoln there was unusual excitement in the female ward. The Commissioners "offer no opinion" as to whether this violence was owing to the absence of mechanical restraint, but they admit there is great want of proper classification at the Lincoln and Suffolk Asylums, to which the scenes they witnessed were partly owing. In offering no opinion, but in so stating the facts as to convey an indirect opinion, we cannot admire either the Commissioners' straightforwardness or wisdom. They saw the whole circumstances, and they were in duty bound to give their conclusions, manfully and decidedly. If they thought that these facts were strong enough to justify the recommendation of moderate restraint, they should have boldly stated it, instead of insinuating it by offering no opinion.

Let us, however, examine the facts. In four asylums containing 1098 lunatics under no restraint, they were all found tranquil and comfortable at both visits. In another, containing 1000 patients, there were, on the first visit, four unruly patients; one of these was so violent as to require removal by manual force and seclusion; two *might* have been prevented by mechanical restraint from injuring themselves; and one from injuring another; but in neither case was any material injury caused. At the second visit all the thousand patients were quiet and comfortable. In two other asylums containing together 502 patients, the women in the refractory wards were violent; but in both institutions *there is a want of proper classification*, and as this is an indispensable part of the system of non-restraint, and is essential to its success, the experiment was not fairly carried out. These facts seem to us to convey the strongest affirmative evidence of the success of non-restraint. Two thousand insane patients are, with four exceptions only, found tranquil and comfortable, who are treated with mild and kind means, without the employment of muffs, strait-waistcoats, restraint chairs, or any other manacles under similar euphonistic titles. That such evidence should not be considered by the Commissioners as worthy of comment proves strikingly how the cause of humanity has advanced since Dr. Conolly had the moral courage to try the non-restraint system on a thousand patients at once.

The other county and public asylums adopt mechanical restraint occasionally; but "the system pursued is that of dispensing with it in every case, unless either the cure or the security of the patient or others is considered to render it necessary." Of course such a rule as this is open to the widest construction; no one who used even the old system of restraint in its whole extent and barbarity, would admit that he was not guided by this rule.

These asylums contain 2868 patients, 24 of whom were under restraint at the time of the Commissioners' visits, a number greater than the proportion of cases in the other asylums, which the Commissioners think *might* have been benefited by mechanical restraint; but yet most satisfactorily small; an improvement which no one can doubt is to be ascribed to the success of the non-restraint system itself, carried out in its whole integrity; an improvement which would never have been effected, had not the system of non-restraint been firmly advocated and perseveringly pursued.

The Commissioners find it more difficult to convey an accurate view of the licensed houses. Of these there are 142, and in two only (Denham Park and Fairford) mechanical restraint is entirely suppressed. "These houses are both well managed." In 60 houses in the provincial district receiving private patients only, and having 702 patients, 15 persons were in restraint. In 32 similar houses in the metropolitan district having 618 patients, 6 only were in restraint. In the establishments at Bethnal Green and Hoxton, containing about 1000 patients, chiefly paupers, about ten or twelve are in restraint at a time.

The Commissioners have entirely omitted to state the number of paupers under restraint in the private asylums in the provinces. As there are 2257 paupers so confined,—as the Commissioners were directed by the Act "to inquire in every licensed house whether any patient is under restraint, and why," this is a serious and unaccountable omission.

The number of paupers under restraint in county asylums is given; and of private patients in private houses, but the number under restraint of that large body of paupers who are consigned to private hands, is not even alluded to; and no reasons are given for this ominous silence. We regret that we have not such tables of the system of restraint in private houses where much of the old treatment of paupers still prevails, in order to compare them with the tables furnished by our non-restraint county asylums. We have no sort of doubt but that the comparison would furnish additional reasons for carrying out the non-restraint system, and supply evidence of the necessity of additional county asylums. We trust that some member of Parliament will move that this deficiency be supplied.

As restraint is going out of fashion, solitary confinement is coming into "more general use. Great numbers of the superintendents of public, and the proprietors of private asylums throughout the country, are fitting up and bringing into use, solitary cells, and padded rooms for violent and unmanageable lunatics." The Commissioners allude to the abuses to which seclusion is subjected, and judiciously recommend that the law should require a register of all cases in which solitary confinement is resorted to.

The next section is headed "Danger of total disuse of mechanical restraint, illustrated by cases." Ten cases are given. In three of these danger was apprehended and restraint used. In four others death, amputation or severe injury followed bites or kicks in asylums where restraint is employed. And in the other three, watching irritated the patients whilst "muffs" or other mechanical restraints "tranquillized." The Commissioners do not seem to have taken into their consideration the important fact that all these cases occurred in asylums where restraint is used, that its employment did not therefore prevent the occurrence of accidents from sudden violence, which happened in four cases. With regard to the three cases where watching irritated and muffs tranquillized, we doubt not that every asylum where restraint is not abolished would supply similar ones. If the watchers do not perform their irksome duty with kindness and consideration they are likely to irritate, and if such attendants who have been used to the much easier process of mechanical restraint can have recourse to it whenever they think it to be necessary, they will very naturally believe it to be necessary, and that the disease and not their own conduct renders it so. The patient is irritated by his attendants until he thinks nothing will quiet him but "muffs," and when these are applied the keeper leaves the poor wretch alone, and he falls asleep. The danger of the total disuse of mechanical restraints should not have been proved from cases occurring in asylums where restraint is employed, but in those where non-restraint is adopted. The reasoning of the Commissioners is vicious, they take for granted that mechanical restraint would have prevented certain injuries, which is the point which was to be proved.

The opinions of the medical superintendents of asylums are generally in accordance with their practice. Those of Lincoln, Northampton, Haslar, Hanwell, and Gloucester, think that mechanical restraint is not necessary or advisable in any case whatever. The superintendents at Lancaster and Suffolk asylums think that cases may occasionally occur which require it. This too is the opinion of the superintendents of the other county asylums, and public hospitals for the insane, 8 of whom think it necessary as a precaution and remedial agent, and 3 that it is less irritating than manual force and seclusion. Such seems to be the opinion of those keeping private houses, with the two exceptions of Denham Park and Fairford.

The Commissioners think it their duty to call attention to the fact that since the autumn of 1842, two of the attendants have been killed, three or four most seriously injured by dangerous patients, some of these injuries happened in asylums where restraint is practised and others where non-restraint. One of the strongest reasons for the non-restraint system is omitted. We quote from the last Report of Bethlem as from a source where no undue partiality can be suspected as occasional restraint is used at that hospital: "The experience of the last year adds another confirmation to the *now generally received opinion* that mechanical restraint is an exciting cause for suicidal propensities; *and though it may for the time* restrain the attempt, it fosters and strengthens the desire it is intended to control."

This chapter on restraint is the least satisfactory one in the Report. The Commissioners lean to the system which admits occasional restraint, but they do not firmly and openly avow this to be their opinion, although they convey this impression indirectly by their mode of stating their facts. It is this apparent want of openness which we object to, not to their opinion differing from our own, although we do not think their view is borne out by a large survey of the whole subject, or even by their own facts. For the question is one regarding the general management of lunatics as a body. Whether, on the whole, 20,000 lunatics would be in most favorable circumstances as to their recovery, bodily comfort, and mental tranquillity, if even subjected to handcuffs, muffs, or chains; or whether this point should be left to the discretion of their attendants. Of course, as in all subjects connected with the regulations of human institutions, there must be a balancing of evils. The question is, on which side the good preponderates over the imperfections to which all our plans are liable, and if in the one place the good does preponderate, and it can be carried out without wrong or injustice to others, however few those may be, we are bound to adopt it, although it may be liable to some objections.

Six years ago the experiment of non-restraint in all cases was tried at Lincoln, and a year subsequently Dr. Conolly, in the face of opposition, contempt, and scepticism, decided on making the bold experiment of abolishing mechanical restraint at once from Hanwell, where there were then 850 pauper lunatics under his care. The experiment, this Report allows, has been eminently successful. It has been followed by Lancaster, Gloucester, Northampton, and Haslar, where non-restraint and kindness have been carried to their fullest extent, and with entire success. The *direct* result has been the increased comfort and tranquillity of a large body of lunatics by the diminution of much unnecessary cruelty and harshness to which they were before subjected, and the *indirect* result is that the law of kindness instead of severity is recognized in a greater extent than formerly in all lunatic asylums (with but few exceptions) and the condition of their unhappy inmates thereby improved. It has not prevented suicides; but from the statistics of Bethlem it is fair to conclude that it has diminished their frequency. It has not prevented accidents from sudden outbreaks of fury, any more than the restraint system; but there is no reason to believe that such have been more numerous. They belong to the nature of the disease, and no one plan but the barbarous one of complete and entire confinement of all who are not idiots or demented would prevent them. The system which the Commissioners advocate does not, for these accidents have occurred chiefly in asylums conducted on their own principle, and have happened also where much more rigorous mechanical restraints have been the rule. But that the non-restraint system is of equal security with the other, both being somewhat insecure, is proved by the evidence of this Report itself. When therefore we take into consideration the notorious abuses of the system of restraint, to which it is so peculiarly liable, and the great fact that by carrying out this new system to an extreme, so much good has been effected without any increase of the inconveniences experienced under the old system; we regret that the Commissioners have not given the more decided sanction of their authority and recommendation to non-restraint, and have not even fully recognized the advantages which it has already conferred, and those to which it must lead.

### VI. RELIGIOUS SERVICES.

The services of the church are for the most part regularly performed every Sunday, and in many cases on other days of the week. The effect is tranquillizing, the patients look forward to the service with pleasure, instances of misconduct during it are very rare. The superintendents all agree as to its soothing effects, but some think that this ceases with the service; all agree that injudicious religious instruction and controversial discourses are injurious. At private asylums the proprietor or superintendent reads the services, some few have engaged the services of a clergyman. Chaplains have been appointed to all the county asylums. At Hanwell the communion is administered to those who wish it, and are considered fit to receive it. The Commissioners recommend that in all cases where it is practicable it is desirable to obtain the services of the incumbent of the parish, or a neighbouring clergyman, and that he should be required to make minutes of his visits, and the numbers attending prayers in a book kept for the purpose.

VII. ADMISSION AND LIBERATION OF PATIENTS.

An order signed by two medical men, each having separately examined the lunatic, is required by law for the admission of a private patient into a licensed house, and signed by one medical man, one justice, or the officiating clergyman and the overseer, for the admission of a pauper. The visitors are required to examine these orders, which they should never neglect to do. Orders for admission into county asylums for private patients must be signed by one medical man, and for a pauper of the same county by two justices, or of another county by one justice. The Commissioners suggest that one form of admission for all private patients, and one for all paupers, would be desirable. The law enacts that it is a misdemeanour to receive any lunatic in any house not licensed, without sending a copy of the order to the clerk of the Metropolitan Commissioners. This is rarely attended to, and the Commissioners state that they have obtained information, from which, there can be no doubt, that pauper lunatics have been, and still are, subject to very severe and unjustifiable restraint, in cases where they are boarded out singly in the houses of persons who receive them for small sums. At the Leicester County Asylum there is a man at work in good bodily health, who had been seven years before his admission chained night and day in a small back room at Peckleton, in the same county! At Plympton is another who had been constantly chained for eleven years, whilst boarding at a private house! Such cases show the importance of carrying out the law, which supposes every pauper to be under proper supervision. There are 3896 pauper lunatics with their friends or elsewhere than in county asylums, workhouses, or licensed houses. The Commissioners have the power of liberating lunatics. They have rarely found any patient who had been in a sane state when first confined, but they have found many cases in which confinement has been too far prolonged, and not unfrequently met with reluctance on the part of the patient's friends, from timidity or wish of concealment; or of the parish officers, on the ground of expense or trouble, to remove the patient. In such cases, and also where the person signing the order is dead or abroad, (such person's consent being required,) the Commissioners have felt the importance and utility of the power vested in them; and some such authority they think should be invested in some persons to ensure the due protection of the subject. When their interference has been necessary they have in the first place, if possible, recommended that the patient should be removed by his friends, against whom he often feels animosity, from their having confined him; and under such circumstances a certain amount of liberty may be at first allowed, and the patient gradually accustomed to his liberty. The Commissioners themselves cannot grant partial liberty. The general principle which has guided them is "that wherever a man of ordinary intellect is able to conduct himself, that he is not likely to do injury in person or property, to himself or others, he is unfit to continue as the inmate of a lunatic asylum." The difficulty of applying this is great. In cases of crime, which are referred to moral insanity, they think that great scrutiny is necessary, and "that the shelter of a lunatic asylum should not be furnished, except upon incontrovertible grounds, to persons *prima facie* liable to be dealt with by the criminal law of the land."

In no case should a patient be liberated, they think, unless examined by those conversant with insanity. The power of liberation invested in the magistrates at the quarter sessions is exercised generally with discretion. They think it questionable whether any lunatic who has been guilty of violence should be released without their own sanction, or that of the visiting justices, or other competent authority.

They have found much difficulty in determining as to the liberation of patients whose insanity has arisen from drunkenness, and who have recovered, and the only reason for whose detention is their liability to resort to their former practices, and to relapse. Their practice has been, if it is a first attack, and the patient is aware of his misconduct, and desires to reform, to liberate him after a short confinement; but after a second attack to confine him for a much longer period.

### VIII. STATISTICS OF INSANITY.

The law requires the return of all lunatics confined in licensed houses and county asylums, and the tables of the Poor Law Commissioners furnish the numbers of all pauper lunatics belonging to unions; but the returns from parishes not included in unions are imperfect; there are no returns of pauper lunatics receiving out-door relief, and there are no returns to be depended on of single patients under certificate in private houses, nor for that larger class of all ranks under the care of relations and guardians, without certificate. Tables have been carefully prepared of the curable and incurable, and of the average per cent. of cures and deaths, of the number of epileptics, idiots, and of homicidal and suicidal patients, of their state, whether married, single, or widowed, or of their condition of life. It appears that the proportion of pauper lunatics to the population is considerably larger in Wales than in England; and that in England and Wales the whole number of females is greater than that of males in proportion to the whole number of each sex.

### IX. CRIMINAL LUNATICS.

Persons who have been acquitted of crimes, or discharged on the ground of insanity, are either sent to Bethlem or to county or private asylums. There are 224 criminal lunatics so confined in England, 76 in county asylums, 61 in licensed houses, and 85 in Bethlem, besides two at Norwich, in the lunatic ward of the union workhouse. Of 137 confined in county asylums and licensed houses, there are —29 for murder, 4 infanticide, 9 stabbing, shooting, and attempting to drown others, 1 manslaughter, 5 arson, 1 detestable crime—making 49. Thus there are 49 out of 137 confined for atrocious offences; and it seems clear that such should not be associated with ordinary lunatics. The others are cases of theft, minor assaults, and other misdemeanours, "to which comparatively little objection can be made to their associating with other patients." When it is considered that there are so many confined for atrocious offences; that although often attempted it is difficult to conceal from the other patients that their associate is a criminal lunatic; that there are no proper wards to prevent escape and that the feelings of the relatives of patients must be outraged on criminals being forced into their society, the commissioners have good reasons for recommending that arrangements should be made for the separate care of this class, either with one or more public institutions (as Bethlem), or that a separate class should be formed in a convenient prison, separate from the other prisoners.

WALES.

The Commissioners conclude their Report by bringing under the Lord Chancellor's special consideration the "very destitute and neglected state of the insane poor in Wales." In 1843 there were 1177 pauper lunatics in Wales, 77 of whom were in English county and licensed asylums, 90 in union workhouses, and 1010 boarded with their friends or elsewhere. There is but one house in Wales licensed to receive 36 pauper patients, besides the old prison at Haverfordwest, which has been so strongly animadverted upon. Mr. Williams, the principal medical practitioner at Denbigh, after enumerating several instances of neglect, says,

"The state of the Welsh lunatic, in general, is most pitiable and miserable,—with no place of protection in his own country; and the only alternative is an English asylum, where no real good can be effected, and that at an enormous expense to his parish or his friends."

One case he mentions of a man (who had been a student at Oxford) who was confined in a small room, unshaven and uncleaned, for 20 years!

The Commissioners were shown a letter addressed by a parish officer to the proprietor of a licensed house. We reprint it, as it speaks volumes:

"Blaenbwch, 19 Dec. 1843.

Sir,—We have got an insane female pauper of the Union of Buillht, Breconshire: she has been so for some years. Something has took her in her limbs about two years ago, until she is quite a cripple. I suppose it is owing to being kept in a close place, having no practice to walk. Please to write me the lowest you charge per week."

We feel that any comment on this is superfluous. "The close place," and "no practice to walk," in which her sad life must have been spent for so long a time,—for years before it crippled her;—the parish official first attributing the loss of her power of motion to an indefinite "something which took her in her limbs," and then his glimmering of common sense that this something was "the close place," and "no practice to walk,"—and then the inquiry as to "the lowest you charge per week," as if the whole had been, and was, a mere question of economy,—and the same "close room" and "no practice to walk" would still be the unhappy woman's lot until death rescued her—if the charge "per week," was not "the lowest;"—is a picture of the misery to which some of the poor are still subjected, on which Crabbe, with his stern truthfulness, might have founded one of his harrowing tales, though he would have been incapable of conveying in the same compass the same truths as this unconscious overseer.

That this misery is not confined to one place, we quote the following from a letter of the Dean of St. Asaph, dated 28th Oct. 1842:

"I have seen one secured in a dark and loathsome shed, lying extended upon straw (for the space did not admit of his standing erect), in a state of filth that I dare not describe. A second was fettered and manacled, and basking in the public street, exposed to the rude gaze of the thoughtless passers by. A third I have seen led about the streets, and even to church, in the restraint of a strait-waistcoat. A fourth (I copy from a letter now before me of a justice of one of the Welsh counties) died some time ago in the most deplorable state, having been, for about fifteen years, chained like a wild beast in an outhouse."

The Commissioners conclude their Report with 25 suggestions for the amendment of the law, all of which, except those which relate to details of no general interest, we have previously alluded to or discussed.

This article has extended itself beyond the limits we had originally intended, from the quantity of valuable matter in the Commissioners' Report, all of which we have laid before our readers in a condensed form. Such a Report may be in parts both erroneous and deficient: on the one side, it may occasionally convey stronger impressions of abuses than the whole exposition of the case would warrant; but, on the other hand, how much of the misery and wretchedness which lunatics suffer from mismanagement, is to the Commissioners as a sealed book. The physical causes of misery, the chains, the dirt, the wretched rooms, all that directly meets the eye may have been observed; but yet, as it is the direct interest of proprietors to conceal any abuses, or to gloze them over, it is not improbable that they have, on the whole, understated these, rather than overstated them. But the mental misery suffered from a sense of degradation, from confinement, and from a want of forbearance, from the insolence and harshness of attendants, is almost wholly concealed. This is the suffering to which the private patients are especially exposed; of those whose will before has been their law; who find themselves at once subjected to the despotic control of others; imprisoned, manacled perhaps, and subjected to the constant society, to the gibes, the coarse jokes, the manifold impertinences of vulgar-minded attendants who keep them in subjection, either by the threats or the application of physical force. This "insolence of office," this close companionship with menial attendants, are the miseries which the educated and refined feel so bitterly, and (as their public and private revelations show,) look back to with so much horror: many of which escape the notice of the medical superintendent, and few of which can be discovered in the short and annual visits of Commissioners, but lie hid in the hearts of those who know their own bitterness. But these are deficiences inherent in the subject itself where the whole truth cannot be known; deficiencies which do not diminish the importance of the partial truths disclosed in this valuable document, which contains information which all should be acquainted with.

We heartily wish that this Report may lead to two grand improvements: one is the appointment of a central body of visiting commissioners empowered to visit annually all the lunatic asylums in the kingdom, and to make an annual report to Parliament upon them. This will be a check upon these abuses, which always do and must attach to the exercise of despotic power, especially when subject to the secondary influence of interest; and as the law gives almost despotic power to superintendents and proprietors of lunatic asylums and governors of workhouses, over 20,000 human beings, it is but just that these individuals, to whom it has delegated such power, should be minutely responsible to its authority, and under its constant and careful supervision. This will be of advantage both to the rich and the poor, as both are subject to the same abuses of misused power. The other desideratum is the general erection of county lunatic asylums. Such asylums should, like our hospitals, be regarded as the proudest monuments of our age,—monuments which will be indications to the future inquirer, that wealth, and commercial greatness with the epicurean selfishness which it tends to foster, had not wholly corrupted England; that she was not alone remarkable for her mechanical arts, for her railroads, steamvessels, and manufactories; that if the age of the beautiful was passed, and she could no longer build the lofty rhyme or the gothic cathedral, or embody ideal beauty on the canvass or in stone, she had at least brought her wealth, her science, her mechanical skill, and general practical knowledge, to alleviate the diseases of the poor, and opening wide her hand, even to prodigality, had over the whole breadth of the land, embodied the divinest of the virtues—Charity.

---

